# The luteinizing hormone beta-subunit exon 3 (Gly102Ser) gene mutation and ovarian responses to controlled ovarian hyperstimulation

**Published:** 2014-10

**Authors:** Robab Davar, Nasim Tabibnejad, Seyed Mehdi Kalantar, Mohammad Hasan Sheikhha

**Affiliations:** *Research and Clinical Center for Infertility, Shahid Sdoughi University of Medical Sciences, Yazd, Iran.*

**Keywords:** *Ovulationinduction*, *LHβgene*, *Singlenucleotidepolymorphisms*

## Abstract

**Background:** Despite extensive progress in IVF techniques, one of the most difficult problems is the variability in the response to controlled ovarian hyperstimulation (COH). Recent studies show the effects of individual genetic variability on COH outcome.

**Objective:** To evaluate the correlation between LHβ G1502A polymorphisms in exon 3 of the LH gene and ovarian response to COH.

**Materials and Methods: **A total of 220 women treated with a long protocol for ovarian stimulation were studied. Three genotypes of GG, GA and AA were detected by polymerase chain reaction-restriction fragment length polymorphism (PCR-RFLP) analysis.

**Results: **In total, 34 (17%) patients were poor responders, 154 (77%) were normal responders and 12 (6%) were hyper responders. The most frequent genotype was GA (55.5%) whereas 44.5% of patients showed GG genotype and there was no patient with AA genotype. In total 54.5% of normal responders, 61.8% of poor responders and 50% of hyper responders showed GA genotype.

**Conclusion:** Our results did not establish a significant relationship between this polymorphism and the ovarian response. Therefore it is still very difficult to use the genotype of patients for prediction of the ovarian response to stimulation.

## Introduction

As the infertility rate has grown, assisted reproductive techniques (ART) have improved to provide more treatment options. Nowadays more couples seeking ART and therefore more effective and less harmful protocols must be developed. Despite extensive progress in ART, the pregnancy rate per initiated ART cycle and the delivery rate are still around 30% (1). One of the most difficult problems of in vitro fertilization (IVF) treatment is the variability in the response to controlled ovarian hyperstimulation (COH) which ranging from poor to high, leading to IVF failure or complications related to ovarian hyperstimulation syndrome (OHSS). 

Implantation failure could be caused by many different factors such as inappropriate ovarian stimulation, suboptimal laboratory culture conditions and embryo transfer (ET) techniques. In addition to these exogenous factors, endogenous parameters such as age, ovarian reserve and hormonal status proposed to determine success of ART treatment. COH effectiveness is an important factor to determine the expected outcome of IVF. There are some predictive markers of COH effectiveness such as maternal age and ovarian reserve, but the optimal predictors are not clear yet because of high variability of clinical outcome of COH in infertile women. It was shown that individual genetic variability has effects on COH outcome (1, 2). 

There are different definitions of normal, poor or hyper responses to ovarian stimulation. Many of these definitions are related to retrieved oocyte number. It is mentioned that ovarian response must be evaluated in the patients with the same stimulation protocol used (2). Based on a definition, poor response to stimulation is the retrieval of fewer than four oocytes in response to an ovarian stimulation protocol while retrieval of more than 15 oocytes indicate hyper response and anything between these ranges is called normal response (3, 4). Follicle stimulating hormone (FSH) and luteinizing hormone (LH) are two members of glycoprotein hormone family which regulate gonadal function and menstrual cycle. FSH and LH are α: β heterodimers in which the α-subunit is common to both and the β-subunit is unique (5). The effect of FSH and LH is applied by their receptors. Recently, genetic variability of these receptors genes and their receptors has been considered as an important predictive marker in response to ovarian stimulation and outcome of ART cycles (6-8). 

LH binds to its cell surface receptor called LHR. LH and human chorionic gonadotropin (hCG) bind to the same receptor, therefore LHR also is known as LHCGR. This receptor is critical for maintenance of the theca, maturation of follicles and ovulation. Some literatures confirm that mutation in the *LHβ* and *LHR* gene may change the function or structure of the LH and LHR, which consequently can activate or inactivate their bioactivity. These changes can cause amenorrhea, hypo-oestrogenism, anovulation and polycystic ovary syndrome (9, 10). 

Single nucleotide polymorphisms (SNPs) are relatively new markers of the human genome which can demonstrate genetic associations of candidate genes to complex disorders such as infertility. Most studies have been focused on FSH receptor and showed that FSH receptor polymorphism is correlated with response to ovarian stimulation (11-13). However the effect of *LHβ* gene variants on ART outcome remains largely unknown. Studying the SNP as a marker of the human genome, should reveal genetic associations of different responses to COH. A number of functional SNPs, affecting the LH bioactivity, have been described in *LHβ* gene. So far, several common polymorphisms have been identified in *LHR* gene including 18insLeuGln, Asn291Ser and Ser312Asn which are associated with increased receptor activity (14, 15). 

One of the reported *LHβ* polymorphisms is a single missense mutation of G1502A in exon 3 of the *LH* gene, causing the amino acid substitution of serine for glycine at amino acid 102 (Gly102Ser). This gene variant had an effect on LH protein and may enhance the LH bioactivity. In fact it was found that carriers of the LHβ 1052A allele had lower LH level and this polymorphism may be implicated in female infertility, possibly endometriosis-associated infertility in some women (16). 

As LH plays central roles in the maturation of the ovarian follicles and ovulation, therefore it is possible that patients with *LHβ* 1052A allele needs more hormonal induction for controlled ovarian stimulation and as a consequences they may be are susceptible to OHSS. Genetic variability is a very important predictor of the response to COH and the success and safety of IVF treatment. The influence of different genes' polymorphisms was investigated on the outcome of COH in IVF. There is not enough evidence about the relationship between *LHβ* polymorphism and COH yet. Therefore, it is still very difficult to predict the accurate prognosis of COH (17). 

In the present study we tried to provide a step towards the prediction of the accurate prognosis of COH by evaluating the correlation between *LHβ* G1502A polymorphisms in exon 3 of the *LH* gene and ovarian response to COH. 

## Materials and methods

**Patients**

In this analytical cross-sectional study, 220 women undergoing IVF or intracytoplasmic sperm injection (ICSI) in the Research and Clinical Center for Infertility, Yazd, Iran in 2012 were recruited. Inclusion criteria were primary or secondary infertility and being IVF or ICSI candidate for the first or second time. Exclusion criteria were previous history of more than two times failed IVF or ICSI. All women were treated for ovarian stimulation routinely with long protocol. Pituitary function was suppressed using daily administration of 0.5 mg buserelin (Suprefact, Aventis, Frankfurt, Germany), started in the luteal phase of menstrual cycle. When the ultrasound showed inactive ovaries, buserelin was reduced to 0.25 mg and continued until the day of hCG administration. COH was initiated with recombinant FSH (Gonal F, Serono, Aubnne, Switzerland) 150 IU/day on the day 2 of menstrual cycle. 

Ovarian response was monitored by serial ultrasound examinations and evaluation of serum E_2_ levels, and then gonadotropin dose adjustments were done as required. hCG (Pregnyl, Organon, Oss, The Netherlands) 10,000 IU was administered when at least two follicles reached a mean diameter of 18 mm. When fewer than two follicles with normal growth pattern were detected in ultrasound, the cycle was cancelled. Transvaginal oocyte retrieval was scheduled 34-36 hours after hCG administration by a 17-gauge needle (Cook, Queensland, Australia) and then IVF or ICSI was performed. Embryos were transferred within 48-72 hr by a Labotect catheter (Labotect GmbH, Gottingen, Germany). 

The luteal phase was supported by inter muscular administration of progesterone in oil, 100 mg daily for 14 days. Chemical pregnancy was evaluated by measuring serum β-hCG level 14 days after embryo transfer. A transvaginal ultrasonography was performed 3 weeks later for documentation of clinical pregnancy according to the presence of gestational sacs and fetal viability. This study was approved by the Ethics Committee of Research and Clinical Center for Infertility, Shahid Sadoughi University of Medical Sciences, Yazd, Iran. A written informed consent was taken from all patients.

**Genetic evaluation**

Each patient was subjected to blood sampling through peripheral venous tapping into an ethylene diamine tetra acetic acid (EDTA) containing tube. Genomic DNA was extracted from peripheral blood using salting out technique. The *LHβ* G1502A polymorphism in exon 3 of the LH gene was assessed by polymerase chain reaction-restriction fragment length polymorphism (PCR-RFLP) analysis. 

PCR reaction mixture contained 0.1 μg of genomic DNA, 0.4 μM of each primer: forward: AGTCTGAGACCTGTGGGGTCAGC TT, reverse: GGAGGATCCGGGTGTCAGGG CTCCA, 1.25u of Taq polymerase, 1.5 mm of MgCl_2_, and 200 μm of dNTP. Following an initial denaturation step at 94^o^C for 5 min, samples were subjected to 30 rounds of PCR as described previously (18). Products were digested at 37^o^C with 10 IU of restriction enzyme Rsal (Promega UK) followed by electrophoresis on 2% agarose gel, and the digested products were identified using ethidium bromide staining. 

**Statistical analysis**

Data were expressed as mean±SEM Statistical analysis was performed using Statistical Package for the Social Sciences (SPSS), version 15. Differences in the pregnancy outcomes and response to stimulation protocol were compared using Chi-square test. Differences between variables in two groups were analyzed using Independent Sample t test for normal distributed variables and Mann-Whitney U test for abnormal distributed variables. P-value<0.05 was considered statistically significant. 

## Results

The mean age and infertility characteristics of participants are shown in table I. Patients were classified into three groups according to the number of mature follicles in the day of the hCG injection; poor responder (retrieval of less than 4 oocytes), hyper responder (retrieval of more than 15 oocytes) and normal responder (retrieval of more than 4 and less than 15 oocytes). Totally, 34 patients (17%) were poor responder, 154 (77%) were normal responder and 12 (6%) were hyper responder. Patients were divided into three genotypes according to the LHβ G1502A polymorphism; GG, GA and AA. The most frequent genotype of LHβ G1502A polymorphism was GA (55.5%) whereas 44.5% of patients showed GG genotype and there was no patient with AA genotype. 

Female age, duration and etiology of infertility were similar in both GA and GG groups. There were no statistical differences between GA and GG groups in basal FSH, basal LH, number of mature follicles, number of oocyte retrieved and number of embryos (Table II). Serum stradiol level on the day of the hCG administration was significantly higher in the GG group compared with GA group (583.48±523.29 vs. 421.09±410.92, p<0.05). 

Clinical pregnancy rate was 34.8% and 26.1% in the GG and GA groups respectively and the difference was not statistically significant. In total 54.5% of normal responders and 61.8% of poor responders showed GA genotype. Hyper responders were distributed equally between GG and GA genotypes (Table III).

**Table I:** Age and infertility characteristics of patients in both groups

**Variables**	**GG**	**GA**	**p** ***-*** **value**
Female age (years)	29.80 ± 5.51	30.08 ± 6.46	0.125*
Duration of infertility (years)	8.16 ± 9.98	7.80 ± 4.79	0.379*
Male factor no.	49 (55.1%)	61 (55%)	0.950**
Tubal	9 (10.1%)	14 (12.6%)	
Ovarian factor	19 (21.3%)	22 (19.8%)	
Unexplained no.	12 (13.5%)	14 (12.6%)	

Data are presented as mean ± SEM.

* p for Mann-Whitney U test

** p for chi square test

**Table II:** ART cycle characteristics of patients in both groups

**Variables**	**GG**	**GA**	**p-value**
Basal FSH (mIU/ml)	7.06 ± 3.72	7.29 ± 3.37	0.321*
Basal LH (mIU/ml)	6.17 ± 4.26	5.82 ± 3.78	0.754*
Serum stradiol (pg/ml)	583.48 ± 523.29	421.09 ± 410.92	0.020*
No. of mature follicles	11.09 ± 6.49	10.09 ± 6.54	0.177*
No. of retrieved oocytes	10.39 ± 6.06	9.38 ± 5.43	0.294*
No. of embryos	5.37 ± 4.63	5.52 ± 4.56	0.722*
Clinical pregnancy	31 (34.8%)	29 (26.1%)	0.119**

Data are presented as mean ± SEM.

FSH: Follicle Stimulation Hormone

LH: Luteinizing Hormone

* p for Mann-Whitney U test

** p for chi square test

**Table III:** Ovarian response to ovarian stimulation in both groups

**Variables**	**GG**	**GA**	**p-value**
Poor responder	13 (38.2%)	21 (61.8%)	0.689
Normal responder	70 (45.5%)	84 (54.5%)	
Hyper responder	6 (50%)	6 (50%)	

Data are presented as percentages.

p for chi square test

## Discussion

Recently, new techniques in ART treatment lead to higher pregnancy rates for infertile couples. Regardless of this improvement, ART treatment is still associated with risks of OHSS in one side and poor response of ovary in the other side (19). These side effects are related to many factors, among them one of the most important factors is patients' genotypes. This indicates the importance of personalized diagnostic and therapeutic approaches to guaranty the efficacy and safety outcomes of COH (20). FSH and LH are pituitary hormones that control estrogen production in the ovary. hCG is the placental homolog of LH that act through the LHR. It is reported that inactivating mutations in *LHβ* and *LHR *genes are associated with primary amenorrhea and anovulation in women (21). 

Different polymorphisms have been described in the* LHβ* and *LHR* genes. It was shown that two of the *LHR* polymorphisms may have functional relevance and may be involved in hormone binding. They are resulting in amino acid changes of Asn to Ser at codon 291 and Ser to Asn at codon 312 in exon 10 of this gene (22). To our knowledge, very few studies so far analyzed *LHβ* variation in IVF-treated women, therefore further studies are clearly needed to investigate the role of *LHβ* polymorphisms in different responses to COH (23). It was shown that *LHβ* gene mutations may change the structure or function of LH, and therefore activate or inactivate its bioactivity. This alteration can cause many gynecological problems such as anovulation, amenorrhea and polycystic ovary in women. In fact, women with *LHβ* mutations often show amenorrhea and infertility (5). Different studies suggested that the presence of *LHβ* G1502A polymorphisms is associated with infertility and endometriosis associated infertility (16, 24, 25).

The aim of the present study was to assess whether *LHβ* G1502A polymorphism is possible susceptibility alleles and determinants of the response of ovary to COH. Our results did not establish a significant relationship between this polymorphism and the ovarian response. There are few other studies investigating the association of *LHβ* and *LHR* gene polymorphisms and COH. Kerkelä *et al* found no association between 18insLeuGln polymorphisms in the coding region of *LHR* and the development of OHSS, but they found slightly higher incidence of the two-amino-acid insertion in the first exon of the *LHR* gene in OHSS patients than in controls (26). Piersma *et al* have shown that the 291Ser *LHR* variant results in increased LHR sensitivity (14).

Maman *et al* investigated the LHR expression patterns in human granulosa cells (GCs) and its correlation to oocyte function. They concludeی that malfunction of oocytes and low fertilization capacity could be related to the overexpression of LHR in cumulus GCs of MII oocytes (27). One study showed that allele 291Ser/Ser was virtually absent in the Chinese and African-American subjects, this could indicate this fact that the *LHR* 291Asn/Ser SNP is a relatively recent Caucasian mutation (14). Recently in a case report it was shown that a novel heterozygous inactivating mutation in exon 1 of the *LHCGR* gene may provide a potential genetic mechanism for the poor oocyte recovery in some IVF cases (28). 

Papamentzelopoulou *et al* investigated correlation between LHR splice variants expression in cumulus cells and ovarian response as well as ART outcome. They suggested that *LHR* gene expression profile can be used as a biomarker in the prediction of ovarian response and pregnancy success (29). Mafra *et al* studied the *LHβ* G1502A polymorphism by RPLP-PCR in women with endometriosis and infertile women without endometriosis and compared it with control group. The frequencies of genotypes GG, GA and AA in the women with endometriosis were 54.6%, 31.8% and 13.6%, respectively, in the infertile women without endometriosis these rates were 52.4%, 38.1% and 9.5%, respectively, and in the control group these rates were 68.9%, 21.5% and 9.6%, respectively. They concluded that infertility and minimal/mild endometriosis-associated infertility are related to *LHβ* G1502A polymorphism (30). 

In another study *LHβ* gene variant G1502A was found to be higher in Singapore Chinese women who had menstrual disorders (31). Finally Liu *et al* reported that the PCOS carriers of *LHβ* 1052A allele had lower LH level and higher fasting glucose level compared to the control group. They concluded that *LHβ* G1052A mutation may contribute to the pathogenesis of PCOS (32).

## Conclusion

In conclusion, we could not find any relationship between the studied SNP and ovarian response to COH. Considering the results of the present study and all the other studies in this regard, it seems that more studies are needed to help the prediction of the response to COH. It is hoped that advances in the development of genetics diagnostic tools, improve our understanding about the difference in the genotype of patients undergoing ART and by identification of these genetic characteristics of each patients, we can predict the ART and select the most appropriate management strategy. 
